# Distributed control for geometric pattern formation of large-scale multirobot systems

**DOI:** 10.3389/frobt.2023.1219931

**Published:** 2023-09-28

**Authors:** Andrea Giusti, Gian Carlo Maffettone, Davide Fiore, Marco Coraggio, Mario di Bernardo

**Affiliations:** ^1^ Department of Electrical Engineering and Information Technology, University of Naples Federico II, Naples, Italy; ^2^ Scuola Superiore Meridionale, Naples, Italy; ^3^ Department of Mathematics and Applications “R. Caccioppoli”, University of Naples Federico II, Naples, Italy

**Keywords:** multiagent systems, pattern formation, distributed control, swarm robotics, collective dynamics

## Abstract

**Introduction:** Geometric pattern formation is crucial in many tasks involving large-scale multi-agent systems. Examples include mobile agents performing surveillance, swarms of drones or robots, and smart transportation systems. Currently, most control strategies proposed to achieve pattern formation in network systems either show good performance but require expensive sensors and communication devices, or have lesser sensor requirements but behave more poorly.

**Methods and result:** In this paper, we provide a distributed displacement-based control law that allows large groups of agents to achieve triangular and square lattices, with low sensor requirements and without needing communication between the agents. Also, a simple, yet powerful, adaptation law is proposed to automatically tune the control gains in order to reduce the design effort, while improving robustness and flexibility.

**Results:** We show the validity and robustness of our approach via numerical simulations and experiments, comparing it, where possible, with other approaches from the existing literature.

## 1 Introduction

Many robotic applications require—or may benefit from—one or more groups of multiple agents to perform a joint task (Shi and Yan, 2021); this is, for example, the case of surveillance (Lopes and Lima, 2021), exploration (Kegeleirs et al., 2021), herding (Auletta et al., 2022) or transportation (Gardi et al., 2022). When the number of agents becomes extremely large, the task becomes a *swarm robotics* problem (Brambilla et al., 2013; Heinrich et al., 2022). Typically, in these problems, it is assumed that the agents are relatively simple, and thus have limited communication and sensing capabilities, and limited computational resources; see, for example the robotic swarms described in Hauert et al. (2009); Rubenstein et al. (2014); Gardi et al. (2022). Sometimes, to cope with such big ensembles, macroscopic methods exploiting partial differential equations can be also suitable (Biswal et al., 2021; Maffettone et al., 2023a, b).

In swarm robotics, typical tasks of interest include *aggregation*, *flocking*, *navigation*, *spatial organisation*, *collaborative manipulation*, and *task allocation* (Brambilla et al., 2013; Bayindir, 2016). Among these, an important subclass of spatial organisation problems is *geometric pattern formation*, where the goal is for the agents to self-organize their relative positions into some desired structure or *pattern*, e.g., arranging themselves to form multiple adjacent triangles or on a lattice. Pattern formation is crucial in many applications (Oh et al., 2017), including sensor networks deployment (Kim et al., 2014; Zhao et al., 2019), cooperative transportation and construction (Rubenstein et al., 2013; Mooney and Johnson, 2014; Gardi et al., 2022), and 2D or 3D exploration and mapping (Kegeleirs et al., 2021) or area coverage (Wang and Rubenstein, 2020). Moreover, the formation of patterns is common in many biological systems where agents, such as cells or microorganisms, form organized geometric structures, e.g., Tan et al. (2022).

There are two main difficulties associated with achieving pattern formation. Firstly, as there are no leader agents, the pattern must emerge by exploiting a control strategy that is the same for all agents, *distributed* and *local* (i.e., each agent can only use information about “nearby” agents). Secondly, the number of agents is large and may change over time; therefore, the control strategy must also be *scalable* to varying sizes of the swarm and *robust* to uncertainties due to its possible variations.

This sets the problem of achieving pattern formation apart from the more classical *formation control* problems (Oh et al., 2015) where agents are typically fewer and have pre-assigned roles within the formation. Moreover, note that geometric formations can also emerge as a by-product of *flocking* algorithms as those described in Olfati-Saber (2006); Wang G. et al. (2022). Nevertheless in such cases often the focus of the control strategy is to achieve coordinated motion rather than desired regular formations to emerge.

To classify existing solutions to pattern formation, we employ the same taxonomy used in Oh et al. (2015), and later extended in Sakurama and Sugie (2021), which is based on the type of information available to the agents. Namely, existing strategies can be classified as being (i) *position-based* when it is assumed agents know their position and orientation and those of their neighbours, in a global reference frame; (ii) *displacement-based* when agents can only sense their own orientation with respect to a global reference direction (e.g., North) and the relative positions of their neighbours; (iii) *distance-based* when agents can measure the relative positions of their neighbours with respect to their local reference frame. In terms of sensor requirements, position-based solutions are the most demanding, requiring global positioning sensors, typically GPS, and communication devices, such as WiFi or LoRa. Differently, displacement-based methods require only a distance sensor (e.g., LiDAR) and a compass, although the latter can be replaced by a coordinated initialisation procedure of all local reference frames (Cortés, 2009). Finally, distance-based algorithms are the least demanding, needing only the availability of some distance sensors.

A pressing open challenge in pattern formation problems is that of devising new local and distributed control strategies that can combine low sensor requirements with consistently high performance. This is crucial in swarm robotics, where it can be generally cumbersome, or prohibitively expensive, to equip all agents with GPS sensors and communication capabilities, e.g., Rubenstein et al. (2014).

## 2 Related work and main contributions

Next, we give a brief overview of the existing literature before expounding our main contributions. Notice that most of these control strategies are based on the use of *virtual forces* (see Khatib, 1985), an approach inspired by Physics, where each agent is subject to virtual forces [e.g., Lennard-Jones and Morse functions (Brambilla et al., 2013; D’Orsogna et al., 2006)] from neighbouring agents, obstacles, and the environment.

### 2.1 Position-based approaches

In Pinciroli et al. (2008), a position-based algorithm was proposed to achieve 2D triangular lattices in a constellation of satellites in a 3D space. This strategy combines global attraction towards a reference point with local interaction among the agents to control both the global shape and the internal lattice structure of the swarm. In Casteigts et al. (2012), a position-based approach was presented that combines the common radial virtual force [also used in Spears et al. (2004), Hettiarachchi and Spears (2005), Torquato (2009)] with a normal force. In this way, a network of connections is built such that each agent has at least two neighbours; then, a set of geometric rules is used to decide whether any or both of these forces are applied between any pair of agents. Importantly, this approach requires the acquisition of positions from two-hop neighbours. In Zhao et al. (2019), a position-based strategy is presented to achieve triangular and square patterns, as well as lines and circles, both in 2D and 3D; the control strategy features global attraction towards a reference point and re-scaling of distances between neighbours, with the virtual forces changing according to the goal pattern. Therein, a qualitative comparison is also provided with the distance-based strategy from Spears et al. (2004), showing more precise configurations and a shorter convergence time, due to the position-based nature of the solution. Finally, a simple position-based algorithm for triangular patterns, based on virtual forces and requiring communication between the agents, is proposed in Trotta et al. (2018) to have unmanned aerial vehicles perform area coverage.

### 2.2 Displacement-based approaches

In Li et al. (2009), a displacement-based approach is presented based on the use of a geometric control law similar to the one proposed in Lee and Chong (2008). The aim is to obtain triangular lattices but small persisting oscillations of the agents are present at steady state, as the robots are assumed to have a constant non-zero speed. In Balch and Hybinette (2000a, b), an approach is discussed inspired by covalent bonds in crystals, where each agent has multiple attachment points for its neighbours. Only starting conditions close to the desired pattern are tested, as the focus is on navigation in environments with obstacles. In Song and O’Kane (2014) the desired lattice is encoded by a graph, where the vertices denote possible *roles* the agents may play in the lattice and edges denote rigid transformations between the local frames or reference of pairs of neighbours. All agents communicate with each other and are assigned a label (or identification number) through which they are organised hierarchically to form triangular, square, hexagonal or octagon-square patterns. Formation control is similarly addressed in Coppola et al. (2019). The algorithm proposed therein is made of a higher level policy to assign positions in a square lattice to the agents, and a lower level control, based on virtual forces, to have the agents reach these positions. The algorithm can be readily applied to the formation of square geometric patterns, but not to triangular ones. Notably, the reported convergence time is relatively long and increase with the number of agents. Finally, a solution to progressively deploy a swarm on a predetermined set of points is presented in Li et al. (2019). The algorithm can be used to perform both formation control and geometric lattice formation, even though the orientation of the formation cannot be controlled. Moreover, this strategy requires local communication between the agents and the knowledge of a common graph associated to the formation.

### 2.3 Distance-based approaches

A popular distance-based approach for the formation of triangular and square lattices, named *physicomimetics*, was proposed in Spears and Gordon (1999) and later further investigated in Spears et al. (2004); Hettiarachchi and Spears (2005). In these studies, triangular lattices are achieved with long-range attraction and short-range repulsion virtual forces only, while square lattices are obtained through a selective rescaling of the distances between some of the agents. The main drawback of the physicomimetics strategy (Spears and Gordon, 1999; Spears et al., 2004; Hettiarachchi and Spears, 2005; Sailesh et al., 2014) is that it can produce the formation of multiple aggregations of agents, each respecting the desired pattern, but with different orientations. Another problem, described in Spears et al. (2004), is that, for some values of the parameters, multiple agents can converge towards the same position and collide.

Similar approaches are also used to obtain triangular lattices when using flocking algorithms (Olfati-Saber, 2006; Wang et al., 2022b, a). An extension to achieve the formation of hexagonal lattices was proposed in Sailesh et al. (2014), but with the requirement of an *ad hoc* correction procedure to prevent agents from remaining stuck in the centre of a hexagon.

In Torquato (2009), an approach exploiting Lennard-Jones-like virtual forces is numerically optimised to locally stabilise a hexagonal lattice. When applied to mobile agents, the interaction law is time-varying and requires synchronous clocks among the agents. A stability proof for the formation of triangular (or 3D lattices) under the effect of virtual forces control algorithm, was recently published in Giusti et al. (2023). In Lee and Chong (2008), a different distance-based control strategy, derived from geometric arguments, was proposed to achieve the formation of triangular lattices. An analytical proof of convergence was given to the desired lattice exploiting Lyapunov methods. Robustness to agents’ failure and the capability of detecting and repairing holes and gaps in the lattice are obtained via an *ad hoc* procedure and verified numerically. A 3D extension was later presented in Lee et al. (2010).

### 2.4 Main contributions

Our main contributions can be listed as follows.

1. We introduce a novel *distributed displacement-based* local control strategy to solve geometric pattern formation problems in swarm robotics that requires no communication among the agents or any need for labelling them. In particular, to achieve triangular and square lattices, we employ two virtual forces controlling the norm and the angle of the agents’ relative position, respectively.
2. We show that the strategy performs significantly better than distance-based algorithms (Spears et al., 2004) when achieving square lattices, in terms of precision and robustness.
3. We propose an adaptive strategy to select the control gains automatically in order for the agents to organize themselves and switch from one desired pattern to another, without the need of off-line tuning of the control gains.
4. We present an exhaustive numerical and experimental validation of the proposed strategy showing its effectiveness even in the presence of actuator constraints and other more realistic effects.

When compared to the control strategies in the existing literature, our approach (i) is able to achieve both triangular and square lattices rather than just triangular ones [e.g., as in Lee and Chong (2008), Casteigts et al. (2012)] (ii) yields more precise and robust square lattices with respect to distance-based algorithms (e.g., Spears et al., 2004; Sailesh et al., 2014), with only a minimal increase in sensor requirements (a compass); and (iii) does not require the more costly sensors and communication devices used for position-based strategies (e.g., Zhao et al., 2019), nor labelling of the agents (Song and O’Kane, 2014; Coppola et al., 2019).

## 3 Mathematical preliminaries

**Notation.** We denote by ‖⋅‖ the Euclidean norm. Given a set B , its cardinality is denoted by |B| . We refer to $R^{2}$ as the *plane*.

### 3.1 Planar swarms

Definition 1 (Swarm). *A (planar) swarm* S:={1,2,…,N} *is a set of* $N\in N_{>0}$ *identical agents that can move on the plane. For each agent* i∈S *,* $x_{i}\left(t\right)\in R^{2}$ *denotes its position in the plane at time* t∈R .

**FIGURE 1:**
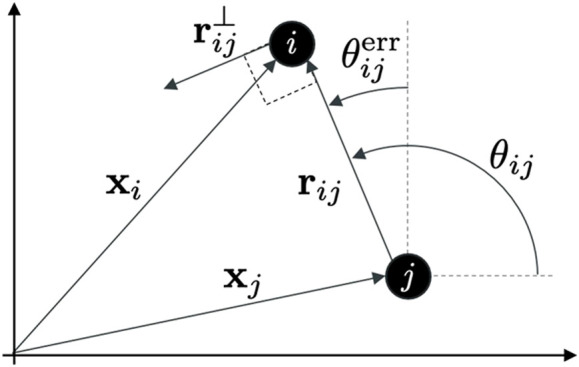
Schematic diagram of two agents, *i* and *j*, showing the key variables used in the paper to describe the agents’ position and their geometrical relationship.

Moreover, $r_{ij}\left(t\right):=x_{i}\left(t\right)-x_{j}\left(t\right)\in R^{2}$ is the relative position of agent *i* with respect to agent *j*, and *θ*_*ij*_(*t*) ∈ [0, 2*π*] is the angle between **r**_*ij*_ and the horizontal axis (see Figure 1).

Definition 2 (Neighbourhood). *Given a swarm and a sensing radius* $R_{\text{s}}\in R_{>0}$ *, the neighbourhood of agent* *i* *at time* *t* *is*

$$N_{i}\left(t\right):=\left\{j\in S⧵\left\{i\right\}:\|r_{ij}\left(t\right)\|\leq R_{\text{s}}\right\}.$$ (1)

**FIGURE 2:**
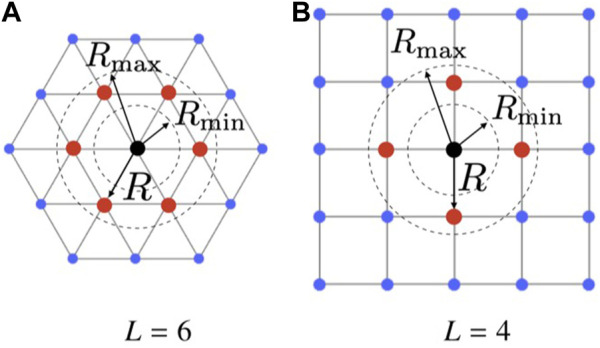
(*L*, *R*)-lattice formations. **(A)** shows a triangular lattice (*L* =6), and **(B)** shows a square lattice (*L* =4). Red dots are agents in the adjacency set $\left(A_{i}\right)$ of the generic agent *i* depicted as a black dot.

Definition 3 (Adjacency set). *Given a swarm and some finite* $R_{\min},R_{\max}\in R_{>0}$ *, with* *R*_min_ ≤ *R*_max_ *, the adjacency set of agent* *i* *at time* *t* *is* (*see* Figure 2).

$$A_{i}\left(t\right):=\left\{j\in S⧵\left\{i\right\}:R_{\min}\leq \|r_{ij}\left(t\right)\|\leq R_{\max}\right\}.$$ (2)

Notice that if *R*_max_ ≤ *R*_s_ then $A_{i}\subseteq N_{i}$ .

Definition 4 (Links). *A link is a pair* (i,j)∈S×S *such that* $j\in A_{i}\left(t\right)$ *(or equivalently* $i\in A_{j}\left(t\right)$ *). Moreover,* E(t) *is the set of all links existing at time* *t*.

Clearly, it is possible to associate to the swarm a time-varying graph G(t)=(S,E(t)) (Latora et al., 2017); S and E(t) being the set of vertices and edges, respectively^1^.

Finally, given any two links (*i*, *j*) and (*h*, *k*), we denote with $\theta_{ij}^{hk}\left(t\right)\in \left[0,2\pi \right]$ the absolute value of the angle between the vectors **r**_*ij*_ and **r**_*hk*_.

### 3.2 Lattice and performance metrics

Definition 5 (Lattice). *Given some* *L* ∈ {4, 6} *and* $R\in R_{>0}$ *, a* (*L*, *R*)-lattice *is a set of points in the plane that coincide with the vertices of an associated regular tiling* (Engel et al., 2004)*;* *R* *is the distance between adjacent vertices and* *L* *is the number of adjacent vertices each point has.*

In Definition 5, *L* = 4, and *L* = 6 correspond to square and triangular lattices,^2^ respectively, as portrayed in Figure 2. We say that a swarm *self-organises into a* (*L*, *R*)*-lattice* if (i) each agent has at most *L* links, and (ii) ∀(i,j)∈E and ∀(h,k)∈E it holds that $\theta_{ij}^{hk}$ is some multiple of 2*π*/*L*. To assess whether a swarm self-organises into some desired (*L*, *R*)-lattice, we introduce the following two metrics.

Definition 6 (Regularity metric). *Given a swarm and a desired* (*L*, *R*)*-lattice, the regularity metric* *e*_*θ*_(*t*) ∈ [0, 1] *is*

$$e_{\theta }\left(t\right):=\frac{L}{\pi }\cdot \theta_{\text{err}}\left(t\right),$$ (3)

where, omitting the dependence on time,

$$\theta_{\text{err}}:=\frac{1}{\left|E{|}^{2}-2|E\right|}\underset{\left(i,j\right)\in E}{\sum }\;\underset{\left(h,k\right)\in E}{\sum }\underset{q\in Z}{\min}\left|\theta_{ij}^{hk}-q\frac{2\pi }{L}\right|.$$ (4)

The regularity metric *e*_*θ*_, derived from Spears et al. (2004), quantifies the incoherence in the orientation of the links in the swarm. In particular, *e*_*θ*_ = 0 when all the pairs of links form angles that are multiples of 2*π*/*L* (which is desirable to achieve the (*L*, *R*)-lattice), while *e*_*θ*_ = 1 when all pairs of links have the maximum possible orientation error, equal to *π*/*L*. (*e*_*θ*_ ≈ 0.5 generally corresponds to the agents being arranged randomly.)

Definition 7 (Compactness metric). *Given a swarm and a desired* (*L*, *R*)*-lattice, the compactness metric* *e*_*L*_(*t*) ∈ [0, (*N* − 1 − *L*)/*L*] *is*

$$e_{L}\left(t\right):=\frac{1}{N}\overset{N}{\underset{i=1}{\sum }}\frac{\left||A_{i}\left(t\right)|-L\right|}{L}.$$ (5)

The compactness metric *e*_*L*_ measures the average difference between the number of neighbours each agent has and the one they are ought to have if they were arranged in a (*L*, *R*)-lattice. According to this definition, *e*_*L*_ reaches its maximum value, *e*_*L*, max_ = (*N* − 1 − *L*)/*L*, when all agents are concentrated in a small region, and links exist between all pairs of agents, while *e*_*L*_ = 1 when all the agents are scattered loosely in the plane, and no links exist between them, and, *e*_*L*_ = 0 when all the agents have *L* links (typically we will require that *e*_*L*_ is below some acceptable threshold, see Section 5.1.1). It is important to remark that, if the number *N* of agents is finite, *e*_*L*_ can never be equal to zero, because the agents on the boundary of the group will always have less than *L* links (Figure 2). This effect gets less relevant as *N* increases. Note that a similar metric was also independently defined in Song and O’Kane. (2014). We remark that the compactness metric inherently penalizes the presence of holes in the configuration and the emergence of detached swarms, as those scenarios are characterized by larger boundaries.

For the sake of brevity, in what follows we will omit dependence on time when that is clear from the context.

## 4 Control design

### 4.1 Problem formulation

Consider a planar swarm S whose agents’ dynamics is described by the first order model

$${\dot{x}}_{i}\left(t\right)=u_{i}\left(t\right),\;\;\forall \;i\in S,$$ (6)

where **x**_*i*_(*t*) was given in Definition 1 and $u_{i}\left(t\right)\in R^{2}$ is some input signal determining the velocity of agent *i* ^3^.

We want to design a *distributed* feedback control law $u_{i}=g\left({\left\{r_{ij}\right\}}_{j\in N_{i}},L,R\right)$ to let the swarm self-organise into a desired triangular or square lattice, starting from any set of initial positions in some disk of radius *r*, while guaranteeing the control strategy to be:

1. *robust* to failures of agents and to noise;
2. *flexible*, allowing dynamic reorganisation of the agents into different patterns;
3. *scalable*, allowing the number of agents *N* to change dynamically.

We will assess the effectiveness of the proposed strategy by using the performance metrics *e*_*θ*_ and *e*_*L*_ introduced above (see Definition 6 and Definition 7).

### 4.2 Distributed control law

To solve this problem we propose a distributed displacement-based control law of the form

$$u_{i}\left(t\right)=u_{\text{r},i}\left(t\right)+u_{\text{n},i}\left(t\right),$$ (7)

where **u**_r,*i*_ and **u**_n,*i*_ are the *radial* and *normal* control inputs, respectively. The two inputs have different purposes and each comprises several *virtual forces*. The radial input **u**_r,*i*_ is the sum of attracting/repelling actions between the agents, with the purpose of aggregating them into a compact swarm, while avoiding collisions. The normal input **u**_n,*i*_ is also the sum of multiple actions, used to adjust the angles of the relative positions of the agents.

Note that the control strategy in (7) is *displacement-based* because it only requires each agent *i* (i) to be able to measure the relative positions of the agents close to it (in the sets $N_{i}$ and $A_{i}$ ), and (ii) to possess knowledge of a common reference direction. Next, we describe in detail each of the two control actions in (7).

### 4.3 Radial interaction control

**FIGURE 3:**
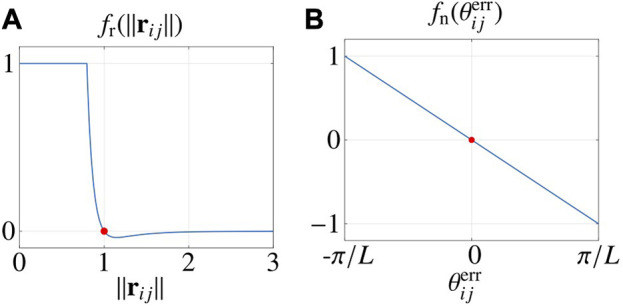
Interaction functions. **(A)** shows the radial function and **(B)** shows the normal interaction function. Red dots highlight zeros of the functions. Parameters are taken from Table 2.

The radial control input **u**_r,*i*_ in (7) is defined as the sum of several virtual forces, one for each agent in $N_{i}$ (neighbours of *i*), each force being attractive (if the neighbour is far) or repulsive (if the neighbour is close). Specifically, we set

$$u_{\text{r},i}=G_{\text{r},i}\underset{j\in N_{i}}{\sum }f_{\text{r}}\left(\left\|r_{ij}\right\|\right)\frac{r_{ij}}{\left\|r_{ij}\right\|},$$ (8)

where $G_{\text{r},i}\in R_{\geq 0}$ is the radial control gain. Note that **u**_r,*i*_ is termed as *radial* input because in (8) the attraction/repulsion forces are parallel to the vectors **r**_*ij*_ (see Figure 1). The magnitude and sign of each of these forces depend on the distance, ‖**r**_*ij*_‖, between the agents, according to the *radial interaction function* $f_{\text{r}}:R_{\geq 0}\to R$ . Here, we select *f*_r_ as the Physics-inspired Lennard-Jones function (Brambilla et al., 2013), given by

$$f_{\text{r}}\left(\left\|r_{ij}\right\|\right)=\min\left\{\left(\frac{a}{\|r_{ij}{\|}^{2c}}-\frac{b}{\|r_{ij}{\|}^{c}}\right),\;1\right\},$$ (9)

where $a,b\in R_{>0}$ and c∈N are design parameters. In (9), *f*_r_ is saturated to 1 to avoid divergence for ‖**r**_*ij*_‖ → 0. *f*_r_ is portrayed in Figure 3A.

### 4.4 Normal interaction

For any link (*i*, *j*), we define the *angular error* $\theta_{ij}^{\text{err}}\in ]-\frac{\pi }{L},\frac{\pi }{L}]$ as the difference between *θ*_*ij*_ and the closest multiple of 2*π*/*L* (see Figure 1), that is,

$$\theta_{ij}^{\text{err}}:=\theta_{ij}-\frac{2\pi }{L}\arg\underset{q\in Z}{\min}\left\{\left|\theta_{ij}-q\frac{2\pi }{L}\right|\right\},$$ (10)

Then, the normal control input **u**_n,*i*_ in (7) is chosen-as

$$u_{\text{n},i}=G_{\text{n},i}\underset{j\in A_{i}}{\sum }f_{\text{n}}\left(\theta_{ij}^{\text{err}}\right)\frac{r_{ij}^{⊥}}{\left\|r_{ij}\right\|},$$ (11)

where $G_{\text{n,i}}\in R_{\geq 0}$ is the normal control gain. Note that each of the normal virtual forces is applied in the direction of $r_{ij}^{⊥}$ , that is the vector normal to **r**_*ij*_, obtained by applying a *π*/2 counterclockwise rotation (see Figure 1). The magnitude and sign of these forces are determined by the *normal interaction function* $f_{\text{n}}:\;\left[-\frac{\pi }{L},\frac{\pi }{L}\right]\to \left[-1,1\right],$ given by

$$f_{\text{n}}\left(\theta_{ij}^{\text{err}}\right)=-\frac{L}{\pi }\;\theta_{ij}^{\text{err}}.$$ (12)

*f*_n_ is portrayed in Figure 3B.

## 5 Numerical validation

In this section, we assess the performance and the robustness of our proposed control algorithm (7) through an extensive simulation campaign. The experimental validation of the strategy is later reported in Section 7. First in Section 5.2, using a numerical optimisation procedure, we tune the control gains *G*_r,*i*_ and *G*_n,*i*_ in (8), (11), as the performance of the controlled swarm strongly depends on these values. Then in Section 5.3, we assess the robustness of the control law with respect to (i) agents’ failure, (ii) noise, (iii) flexibility to pattern changes, and (iv) scalability. Finally in Section 5.4, we present a comparative analysis of our distributed control strategy and other approaches previously presented in the literature. The simulations and experiments performed in this and the next Sections are summarised in Table 1.

**TABLE 1:** List of simulations and experiments reported in this paper with indication of the section and figures were the results are presented.

Scenario	Section	Figure
*Control law* (7),(8),(11)		
Tuning	5.2	4
Validation	5.2	5
Robustness to faults	5.3.1	6
Robustness to noise	5.3.2	7
Flexibility	5.3.3	8
Scalability	5.3.4	9
Comparison with established algorithm	5.4	10
*Adaptive gain tuning* (22)		
Validation	6	11
Robustness analysis	6.1	12
Robotarium experiment	7	13

### 5.1 Simulation setup

We consider a swarm consisting of *N* = 100 agents (unless specified differently). To represent the fact that the agents are deployed from a unique source (as typically done in the literature, see e.g., Spears et al. (2004), their initial positions are drawn randomly with uniform distribution from a disk of radius *r* = 2 centred at the origin^4,^ ^5^.

Initially, for the sake of simplicity and to avoid the possibility of some agents becoming disconnected from the group, we assume that *R*_s_ in (1) is large enough so that

$$\forall i\in S,\;\forall t\in R_{\geq 0},\;N_{i}\left(t\right)=S\backslash \left\{i\right\};$$ (13)

i.e., any agent can sense the relative position of all others. Later, in Section 5.3, we will drop this assumption and show the validity of our control strategy also for smaller values of *R*_s_. All simulation trials are conducted in Matlab ^6^, integrating the agents’ dynamics using the forward Euler method with a fixed time step Δ*t* > 0. Moreover, the speed of the agents is limited to *V*_max_ > 0. The values of the parameters used in the simulations are reported in Table 2.

**TABLE 2:** Simulation parameters.

Parameter	Description	Value
*R*	Desired link length	1 m
*R*_min_	Minimum link length	0.6 m
*R*_max_	Maximum link length	1.1 m
*V*_max_	Maximum speed	5 m/s
*t*_max_	Maximum simulation time	200 s
Δ*t*	Integration step	0.01 s
*T*_w_	Time window	10 s
*a*	Radial interaction function *f*_r_ (⋅)	0.15
*b*	”	0.15
*c*	”	5

#### 5.1.1 Performance evaluation

To assess the performance of the controlled swarm, we exploit the metrics *e*_*θ*_ and *e*_*L*_ given in Definition 6 and Definition 7. Namely, we select empirically the thresholds $e_{\theta }^{*}=0.2$ and $e_{L}^{*}=0.3$ , which are associated to satisfactory compactness and regularity of the swarm. Then, letting *T*_w_ > 0 be the length of a time window, we say that *e*_*θ*_ is at *steady-state* from time *t*′ = *k*Δ*t* (for k∈Z ) if

$$\left|e_{\theta }\right.\left(t^{\prime }\right)-e_{\theta }\left(t^{\prime }-j\Delta t\right)\left|\leq 0.1\;e_{\theta }^{*},\right.\;\forall j\in \left\{1,2,\ldots ,\left\lfloor \frac{T_{\text{w}}}{\Delta t}\right\rfloor \right\}.$$ (14)

We give an analogous definition for the steady state of *e*_*L*_ (using $e_{L}^{*}$ rather than $e_{\theta }^{*}$ ). Then, we say that in a trial the swarm *achieved steady-state* at time *t*_ss_ if there exists a time instant such that both *e*_*θ*_ and *e*_*L*_ are at steady state, and *t*_ss_ is the smallest of such time instants. Moreover, we deem the trial *successful* if $e_{\theta }\left(t_{\text{ss}}\right)<e_{\theta }^{*}$ and $e_{L}\left(t_{\text{ss}}\right)<e_{L}^{*}$ . If in a trial steady-state is not reached in the time interval [0, *t*_max_], the trial is stopped (and deemed unsuccessful). We define

$$e_{\theta }^{\text{ss}}:=\left\{\begin{array}{c} e_{\theta }\left(t_{\text{ss}}\right),\;\;\text{if steady state is achieved},\\ e_{\theta }\left(t_{\text{max}}\right),\;\text{otherwise}. \end{array}\right.$$ (15)

$$e_{L}^{\text{ss}}:=\left\{\begin{array}{c} e_{L}\left(t_{\text{ss}}\right),\;\;\text{if steady state is achieved},\\ e_{L}\left(t_{\text{max}}\right),\;\text{otherwise}. \end{array}\right.$$ (16)

Finally, to asses how quickly the pattern is formed, we define.

$$\;T_{\theta }:=\min\left\{t^{\prime }\in R_{\geq 0}:\;e_{\theta }\left(t^{\prime }\right)\leq e_{\theta }^{*},\;\;\forall t\geq t^{\prime }\right\},$$ (17)

$$T_{L}:=\min\left\{t^{\prime \prime }\in R_{\geq 0}:\;e_{L}\left(t^{\prime \prime }\right)\leq e_{L}^{*},\;\;\forall t\geq t^{\prime \prime }\right\},$$ (18)

$$T:=\max\left\{T_{\theta },T_{L}\right\}.$$ (19)

### 5.2 Tuning of the control gains

For the sake of simplicity, in this section we assume that *G*_r,*i*_ = *G*_r_ and *G*_n,*i*_ = *G*_n_, for all i∈S ; later, in Section 6, we will present an adaptive control strategy allowing each agent to independently vary online its own control gains. To select the values of *G*_r_ and *G*_n_ giving the best performance in terms of regularity and compactness, we conducted an extensive simulation campaign and evaluated, for each pair (*G*_r_, *G*_n_) ∈ {0, 1, *…*, 30}×{0, 1, *…*, 30}, the following cost function, averaged over 30 trials, starting with random initial conditions:

$$C\left(e_{\theta }^{\text{ss}},e_{L}^{\text{ss}}\right)={\left(\frac{e_{\theta }^{\text{ss}}}{e_{\theta }^{*}}\right)}^{2}+{\left(\frac{e_{L}^{\text{ss}}}{e_{L}^{*}}\right)}^{2}.$$ (20)

**FIGURE 4:**
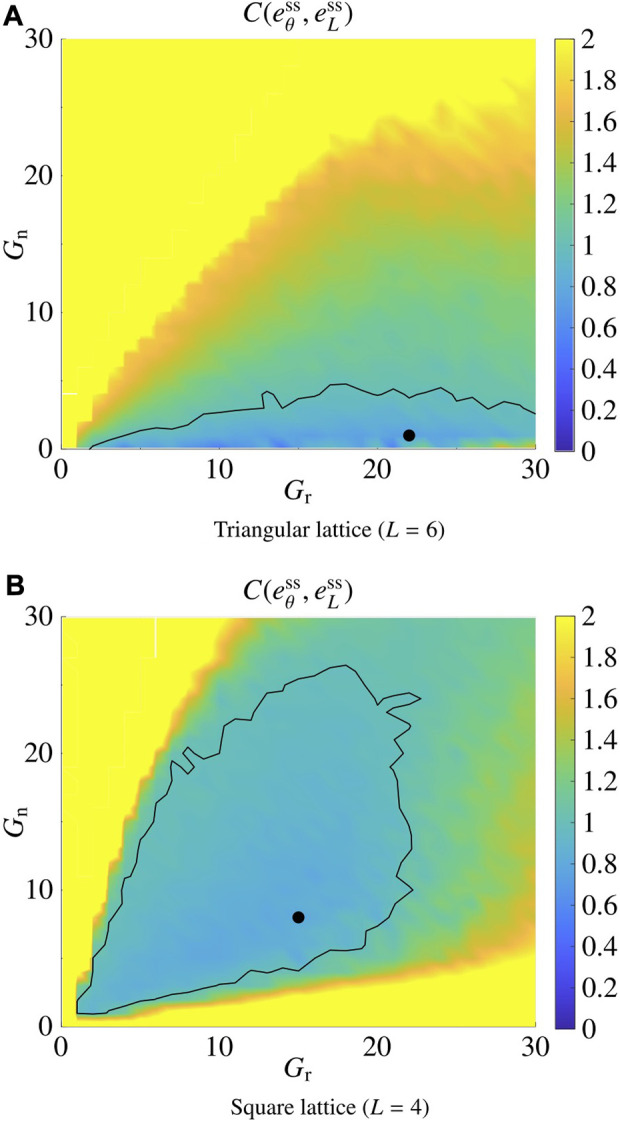
Tuning of the control gains *G*_r_ and *G*_n_. **(A)** shows the result for the triangular lattice (*L* =6), and **(B)** shows the result for the square lattice (*L* =4). The black dots correspond to ${\left(G_{\text{r}}^{*},G_{\text{n}}^{*}\right)}_{L=6}$ and ${\left(G_{\text{r}}^{*},G_{\text{n}}^{*}\right)}_{L=4}$ , minimising the metric *C* defined in (20). The black curves delimit the regions where *C* ≤1.

The results are reported in Figure 4 for the triangular (*L* = 6) and the square (*L* = 4) lattices; in the former case, the pair ${\left(G_{\text{r}}^{*},G_{\text{n}}^{*}\right)}_{L=4}$ minimising *C* is (22, 1), whereas in the latter case it is ${\left(G_{\text{r}}^{*},G_{\text{n}}^{*}\right)}_{L=6}=\left(15,8\right)$ . Both pairs achieve *C* ≤ 1, implying $e_{\theta }^{\text{ss}}\leq e_{\theta }^{*}$ and $e_{L}^{\text{ss}}\leq e_{L}^{*}$ .

**FIGURE 5:**
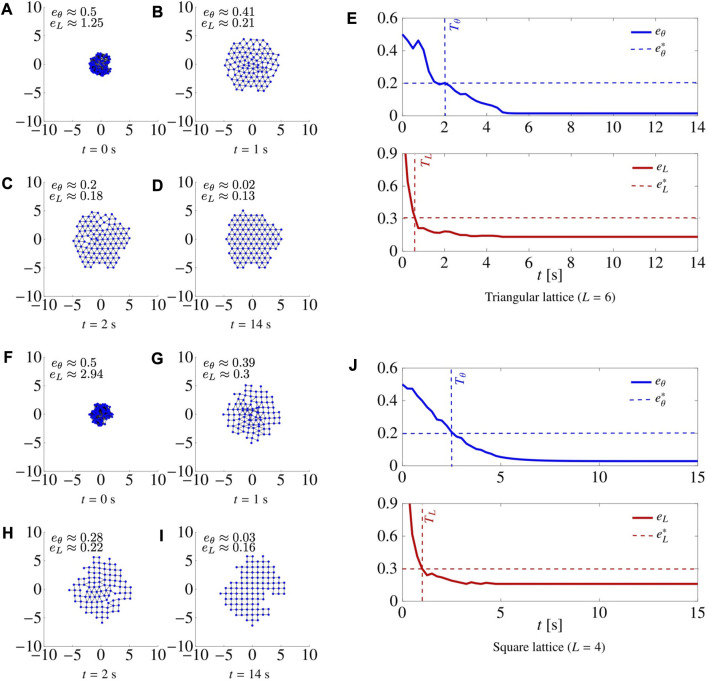
Snapshots at different time instants of a swarm of *N* =100 agents being controlled to form a triangular lattice **(A–D)** and a square lattice **(F–I)**. For each snapshot, we also report the values of *e*_*θ*_ and *e*_*L*_. **(E–J)** show the time evolution of the metrics *e*_*θ*_ and *e*_*L*_ for *L* =6 and *L* =4, respectively. When *L* =6, we set $\left(G_{\text{r}},G_{\text{n}}\right)={\left(G_{\text{r}}^{*},G_{\text{n}}^{*}\right)}_{L=6}$ ; when *L* =4, we set $\left(G_{\text{r}},G_{\text{n}}\right)={\left(G_{\text{r}}^{*},G_{\text{n}}^{*}\right)}_{L=4}$ . (See Section 5.2 for details on how the gains were tuned).

In Figure 5, we report four snapshots at different time instants of two representative simulations, together with the metrics *e*_*θ*_(*t*) and *e*_*L*_(*t*), for the cases of a triangular and a square lattice, respectively. The control gains were set to the optimal values ${\left(G_{\text{r}}^{*},G_{\text{n}}^{*}\right)}_{L=6}$ and ${\left(G_{\text{r}}^{*},G_{\text{n}}^{*}\right)}_{L=4}$ . In both cases, the metrics quickly converge below their prescribed thresholds, as *T* <2.75s . Moreover, note that *e*_*L*_(*t*) decreases faster than *e*_*θ*_(*t*), meaning that the swarm tends to first reach the desired level of compactness and then agents’ positions are rearranged to achieve the desired pattern. Finally, we note that it is immediate to verify that it is possible to control the orientation of the resulting lattice simply by applying a uniform offset to the agents’ compasses.

### 5.3 Robustness analysis

In this section, we investigate numerically the properties that we required in Section 4.1, that is robustness to faults and noise, flexibility, and scalability.

#### 5.3.1 Robustness to faults

**FIGURE 6:**
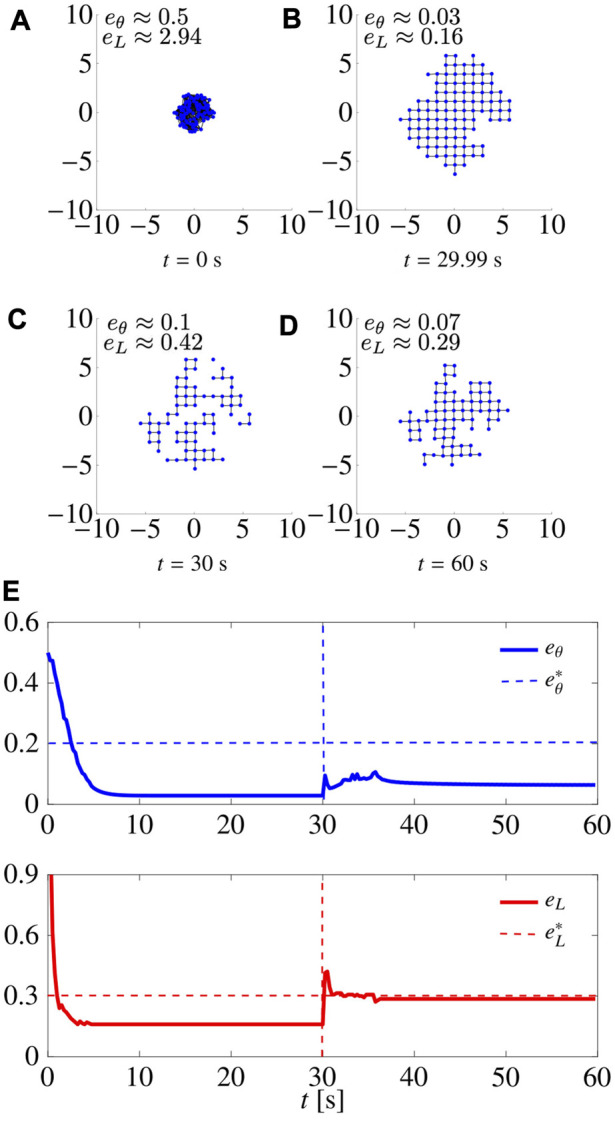
Robustness to agents’ removal. **(A–D)** show snapshots at different time instants of a swarm agents achieving a square lattice. Initially, there are *N* =100 agents with 30 agents being removed at *t* =30 s. **(E)** shows the time evolution of the metrics; dashed vertical lines denote the time instant when agents are removed. Here *L* =4, and $\left(G_{\text{r}},G_{\text{n}}\right)={\left(G_{\text{r}}^{*},G_{\text{n}}^{*}\right)}_{L=4}$ .

We ran a series of simulations in which we removed a percentage of the agents at a certain time instant, and assessed the capability of the swarm to recover the desired pattern. For the sake of brevity, we report only one of them as a representative example in Figure 6, where, with *L* = 4, 30% of the agents were removed at random at time *t* = 30 s. We notice that, as the agents are removed, *e*_*L*_(*t*) and *e*_*θ*_(*t*) suddenly increase, but, after a short time, they converge again to values below the thresholds, recovering the desired pattern, despite the formation of small holes in the pattern at steady-state that increase $e_{L}^{\text{ss}}$ . Finally, we also considered the case where the faulty agents stay still in their positions after the fault, with other agents having to form the lattice around them. We observed that when the fault takes place after a satisfying structure is formed, the metrics are not affected by the event (the numerical results are omitted here for the sake of brevity).

#### 5.3.2 Robustness to noise

We assessed the robustness to noise both on actuation and on sensing, in two separate tests. In the first case, we assumed that the dynamics (6) of each agent is affected by additive white Gaussian noise with standard deviation *σ*_a_. In the second case, we assumed that, for each agent, both the distance measurements ‖**r**_*ij*_‖ in (8) and the angular measurements $\theta_{ij}^{\text{err}}$ in (11) are affected by additive white Gaussian noise (i.i.d. for each *i* and *j*) with standard deviation *σ*_m_ and $\sigma_{m}\frac{\pi }{L}$ , respectively.

**FIGURE 7:**
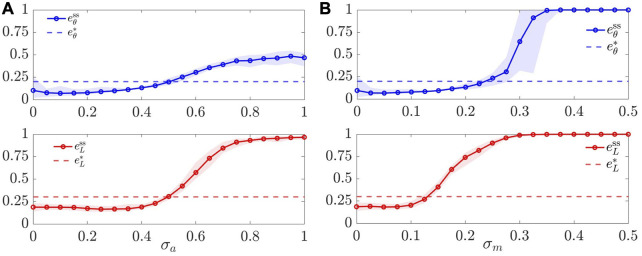
Robustness to noise. Value of the metrics $e_{\theta }^{\text{ss}}$ and $e_{L}^{\text{ss}}$ , averaged over *M* =30 trials, when **(A)** the intensity *σ*_a_ of the actuation noise is varied and **(B)** the intensity *σ*_m_ of the measurement noise is varied. The shaded areas represent the maximum and minimum values obtained over the *M* trials. Here *L* =4, and $\left(G_{\text{r}},G_{\text{n}}\right)={\left(G_{\text{r}}^{*},G_{\text{n}}^{*}\right)}_{L=4}$ .

In particular, we set *L* = 4 and varied either *σ*_a_ or *σ*_m_ in intervals of interest with small increments. For each value of *σ*_a_ and *σ*_m_, we ran *M* = 30 trials, starting from random initial conditions, and report the average values of $e_{\theta }^{\text{ss}}$ and $e_{L}^{\text{ss}}$ in Figure 7. We observe that, while in the ranges *σ*_a_ ∈ [0, 0.45] and *σ*_m_ ∈ [0, 0.125] the strategy guarantees robustness, for large enough noise (*σ*_a_ ≥ 0.45 or *σ*_m_ ≥ 0.125) performance is increasingly worsened with trials eventually becoming unsuccessful (the swarm never achieving the desired lattice configuration). Interestingly, we find that for smaller noise (0 < *σ*_a_ ≤ 0.2 or 0 < *σ*_m_ ≤ 0.1) performance is actually improved, as small random inputs can prevent the agents from getting stuck in undesired configurations, including those containing holes.

We obtained qualitatively similar results when we assumed the presence of noise on the compass measurements of the agents (obtained by adding Gaussian noise on the variables $\theta_{ij}^{\text{err}}$ , with the noise value being the same for $\theta_{ij}^{\text{err}}$ and $\theta_{kl}^{\text{err}}$ when *i* = *k*).

#### 5.3.3 Flexibility

**FIGURE 8:**
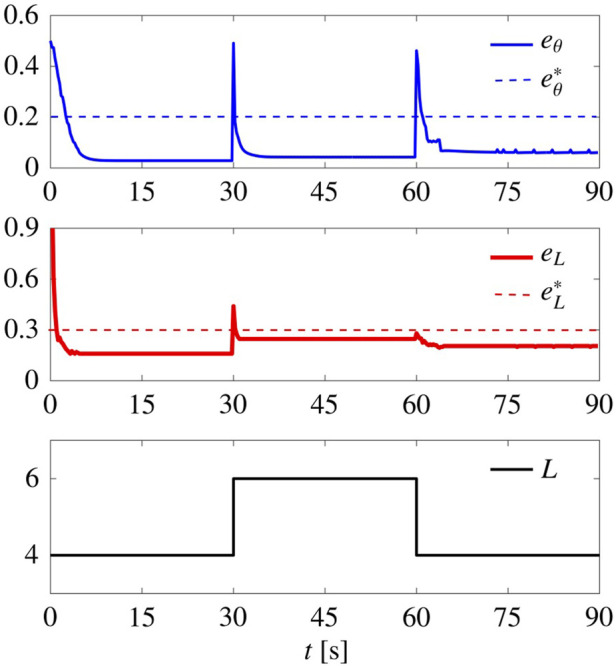
Flexibility to spatial reorganisation. Time evolution of the metrics $e_{\theta }^{\text{ss}}$ and $e_{L}^{\text{ss}}$ as *L* changes as shown in the bottom panel. The gains are set as $\left(G_{\text{r}},G_{\text{n}}\right)={\left(G_{\text{r}}^{*},G_{\text{n}}^{*}\right)}_{L=4}$ .

In Figure 8, we report a simulation where *L* was initially set equal to 4 (square lattice), changed to 6 (triangular lattice) at time *t* = 30 s, and finally changed back to 4 at *t* = 60 s. The control gains are set to ${\left(G_{\text{r}}^{*},G_{\text{n}}^{*}\right)}_{L=4}$ and kept constant during the entire the simulation. Clearly, as *L* is changed, both *e*_*L*_ and *e*_*θ*_ suddenly increase, but the swarm is quickly able to reorganise and reduce them below their prescribed thresholds in less than 5 s, thus achieving the desired patterns.

#### 5.3.4 Scalability

**FIGURE 9:**
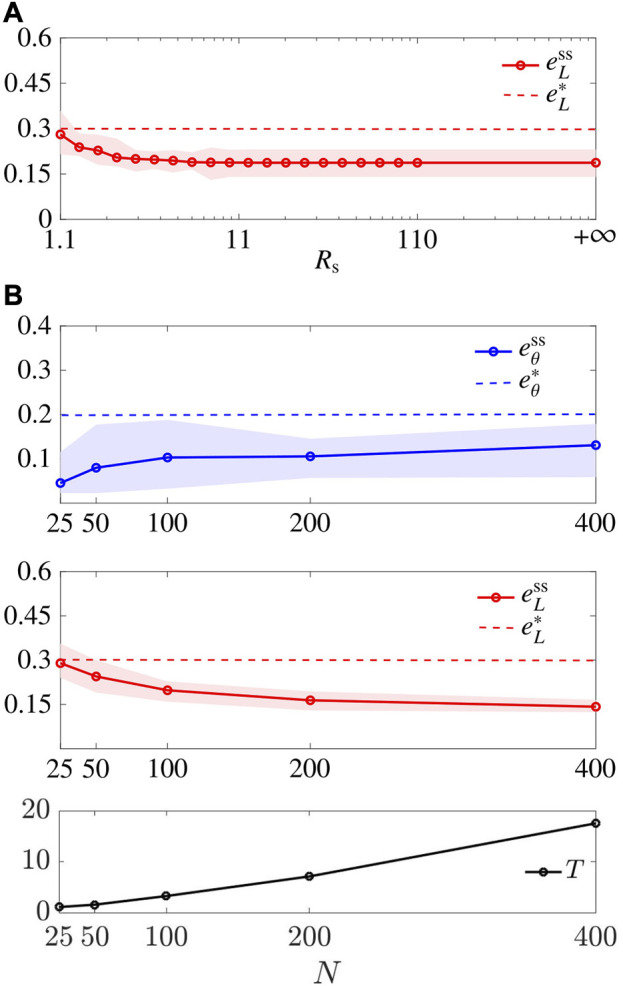
Scalability. **(A)** shows $e_{L}^{\text{ss}}$ averaged over *M* =30 trials for different values of *R*_s_. **(B)** shows the metrics $e_{\theta }^{\text{ss}}$ and $e_{L}^{\text{ss}}$ and the convergence time *T* averaged over the trials, with varying *N*, while *R*_s_ =3 m, and agents’ initial positions are drawn with uniform distribution from a disk with radius $r=\sqrt{N/25}$ . The shaded areas represent the maximum and minimum values over the *M* trials. Here *L* =4 and the gains are set as $\left(G_{\text{r}},G_{\text{n}}\right)={\left(G_{\text{r}}^{*},G_{\text{n}}^{*}\right)}_{L=4}$ .

We relaxed the assumption that (13) holds and characterised $e_{L}^{\text{ss}}$ as a function of the sensing radius *R*_s_. The results are portrayed in Figure 9A, showing that the performance starts deteriorating for approximately *R*_s_ < 6 *R*, until it becomes unacceptable for about *R*_s_ < 1.1 *R*. Therefore, as a good trade-off between performance and feasibility, we set *R*_s_ = 3 *R*. Then, to test for scalability, we varied the number *N* of agents (initially, *N* = 100), reporting the results in Figure 9B. We see that (i) the controlled swarm correctly achieves the desired pattern for at least four-fold changes in the size of the swarm, (ii) compactness $\left(e_{L}^{\text{ss}}\right)$ improves as *N* increases, and (iii) the average convergence time *T* increases as *N* increases.

### 5.4 Comparison with other established algorithm

As done in related literature (Zhao et al., 2019) (yet for a position-based solution) we compared our control law (7) to the so-called “gravitational virtual forces strategy” (see the Appendix) (Spears et al., 2004), that represent an established solution to geometric pattern formation problems. In Spears et al. (2004), a second order damped dynamics is considered for the agents. Hence, for the sake of comparison, we reduced the model therein to the first order model in (6), by assuming that the viscous friction force is significantly larger than the inertial one.

**FIGURE 10:**
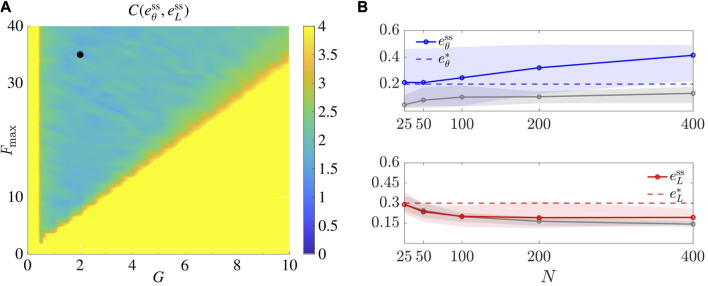
Representative application of the algorithm from Spears et al. (2004) (see Appendix). **(A)** shows the tuning of parameters *G* and *F*_max_ for the square lattice (*L* =4). The black dot denotes the optimal pair $\left(G^{*},F_{\max}^{*}\right)$ . **(B)** shows the scalability test for the algorithm. The metrics $e_{L}^{\text{ss}}$ and $e_{\theta }^{\text{ss}}$ are averaged over *M* =30 trials, as *N* varies, and plotted against our results (in gray) in the same scenario (see Figure 9 for the sake of comparison). Agents’ initial positions are drawn with uniform distribution from a disk of radius $r=\sqrt{N/25}$ . The shaded area represents the maximum and minimum values over the trials. Here *L* =4, and $\left(G,F_{\max}\right)=\left(G^{*},F_{\max}^{*}\right)$ .

To select the gravitational gain *G* and the saturation value *F*_max_ in the control law from Spears et al. (2004), we applied the same tuning procedure described in Section 5.2. In particular, we considered (*G*, *F*_max_) ∈ {0, 0.5, *…*, 10}×{0, 1, *…*, 40}, and performed 30 trials for each pair of parameters, obtaining as optimal pair for the square lattice $\left(G^{*},F_{\max}^{*}\right)=\left(35,2\right)$ (see Figure 10A). All other parameters where kept to the default values in Table 2.

Then, we performed the same scalability test in Section 5.3.4 and report the results in Figure 10B. Remarkably, by comparing these results with ours, we see that our proposed control strategy performs better, obtaining much smaller values of $e_{\theta }^{\text{ss}}$ , regardless of the size *N* of the swarm. In particular, the control law from Spears et al. (2004) only rarely achieves $e_{\theta }^{\text{ss}}\leq e_{\theta }^{*}$ , implying a low success rate.

## 6 Adaptive tuning of control gains

Tuning the control gains (here *G*_r,*i*_ and *G*_n,*i*_) can in general be a tedious and time-consuming procedure. Therefore, to avoid it, we propose the use of a simple, yet effective adaptive control law, that might also improve the robustness and flexibility of the swarm. Specifically, for the sake of simplicity, *G*_r,*i*_ is set to a constant value *G*_r_ for all the swarm, while each agent computes its gain *G*_n,*i*_ independently, using only local information. Letting *e*_*θ*,*i*_ ∈ [0, 1] be the *average angular error* for agent *i*, given by

$$e_{\theta ,i}:=\frac{L}{\pi }\frac{1}{\left|A_{i}\right|}\underset{j\in A_{i}}{\sum }|\theta_{ij}^{\text{err}}|,$$ (21)

*G*_n,*i*_ is varied according to the law.

$$\frac{d}{dt}G_{n,i}\left(t\right)=\left\{\begin{array}{c} \alpha \;\left(e_{\theta ,i}\left(t\right)-e_{\theta }^{*}\right),\;\text{if}\;e_{\theta ,i}\left(t\right)>e_{\theta }^{*},\\ 0,\;\text{otherwise}. \end{array}\right.$$ (22a)

$$G_{\text{n},i}\left(0\right)=0,$$ (22b)

where *α* > 0 is an adaptation gain and $e_{\theta }^{*}$ (introduced in Section 5.1) is used to determine the amplitude of the dead-zone. Here, we empirically choose *α* = 3. To evaluate the effect of the adaptation law, we also define the average normal gain of the swarm ${\bar{G}}_{\text{n}}\left(t\right):=\frac{1}{N}\sum_{i=1}^{N}G_{\text{n},i}\left(t\right)$ .

**FIGURE 11:**
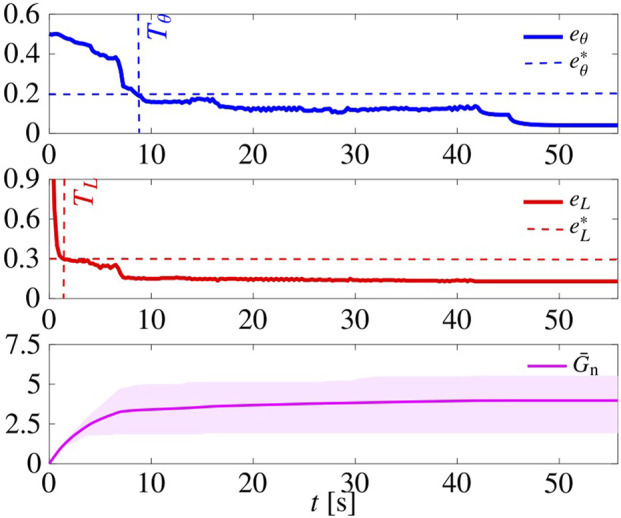
Pattern formation using the adaptive tuning law (22). Initial conditions are the same as those of the simulation in Figure 5. The shaded magenta area is delimited by $\max_{i\in S}G_{n,i}$ and $\min_{i\in S}G_{n,i}$ , while the average across all agents is depicted by a solid magenta line. Here, *L* =4, and *G*_r_ =15.

In Figure 11, we report the time evolution of *e*_*L*_, *e*_*θ*_, and of ${\bar{G}}_{\text{n}}$ for a representative simulation. First, we notice that the average normal gain ${\bar{G}}_{\text{n}}$ eventually settles to a constant value. Moreover, comparing the results with the case in which the gains *G*_n,*i*_ are not chosen adaptively (see Section 5.2; Figure 5J), here *T*_*θ*_, *T*_*L*_ and *t*_ss_ are larger (meaning longer convergence time) but $e_{\theta }^{\text{ss}}$ and $e_{L}^{\text{ss}}$ are smaller (meaning better regularity and compactness performance).

### 6.1 Robustness analysis

Next, we test robustness to faults, flexibility, and scalability for the adaptive law (22), similarly to what we did in Section 5.3.

**FIGURE 12:**
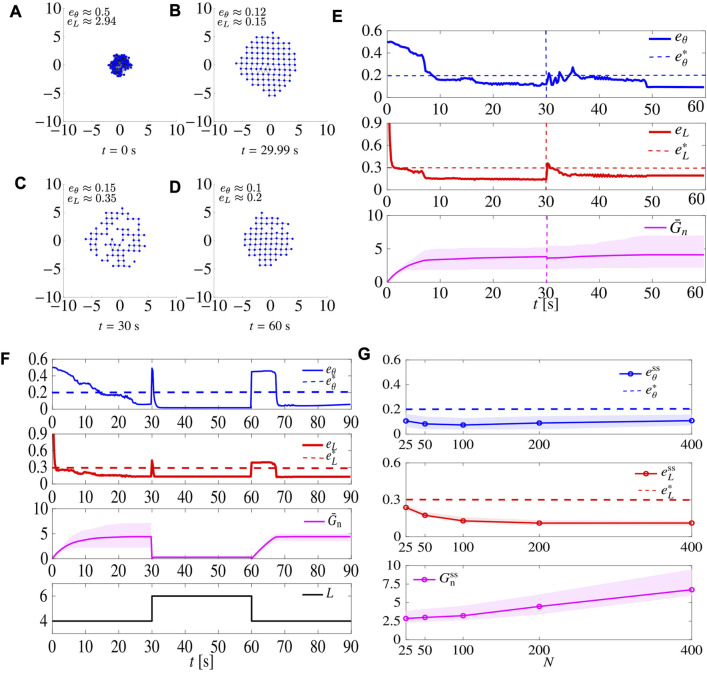
Robustness tests using the adaptive tuning law (22). Panels **(A–E)** show the results of the simulations when starting from 100 agents, 30 agents are removed at *t* =30 s. Initial conditions are the same as those of the simulation in Figure 6. **(A–D)** show snapshots of the agents’ configurations at different time instants. **(E)** shows the time evolution of the metrics *e*_*θ*_ and *e*_*L*_, and of the adaptive gain *G*_n_ (the shaded magenta area is delimited by $\max_{i\in S}G_{n,i}$ and $\min_{i\in S}G_{n,i}$ while the average value of the gain over all the agents is shown as a solid magenta line). Dashed vertical lines denote the time instant when agents are removed. Here *L* =4. **(F)** refers to the flexibility test. Initial conditions are the same as those of the simulation in Figure 8. **(G)** refers to the scalability test. The metrics $e_{\theta }^{\text{ss}}$ and $e_{L}^{\text{ss}}$ , and the adaptive gain $G_{\text{n}}^{\text{ss}}:={\bar{G}}_{\text{n}}\left(t_{\text{ss}}\right)$ are averaged over *M* =30 trials with varying *N*. Agents’ initial positions are drawn with uniform distribution from a disk with radius $r=\sqrt{N/25}$ . The shaded areas represent the maximum and minimum values over the trials. Here *L* =4, and *G*_r_ =15, *R*_s_ =3 m. In all these simulations *G*_r_ =15.

We ran a series of agent removal tests, as described in Section 5.3.1. For the sake of brevity, we report the results of one of such tests with *L* = 4 in Figures 12A–E. At *t* = 30 s, 30% of the agents are removed; yet, after a short time the swarm reaggregates to recover the desired lattice.

We then repeated the test in Section 5.3.3, with the difference that this time we set *G*_r_ = 18.5 (that is the average between the optimal gain for square and triangular patterns), and set *G*_n,*i*_ according to law (22), resetting all *G*_n,*i*_ to 0 when *L* is changed. The results are shown in Figure 12F. When compared to the non-adaptive case (Figure 8), here $e_{\theta }^{\text{ss}}$ and $e_{L}^{\text{ss}}$ are smaller (better pattern formation), but *T*_*θ*_ and *T*_*L*_ are larger (slower), especially when forming square patterns. Interestingly, when *L* = 4, ${\bar{G}}_{n}$ settles to about 5, while when *L* = 6 it settles to about 0.3, a much smaller value.

Finally, we repeated the test in Section 5.3.4, setting again the sensing radius *R*_s_ to 3 *R* and assessing performance while varying the size *N* of the swarm; results are shown in Figure 12G. First, we notice that the larger the swarm is, the larger the steady state value of ${\bar{G}}_{n}$ is. Comparing the results with those obtained for static gains shown in Figure 9B, here we observe a slight improvement of performance, with a slightly smaller $e_{\theta }^{\text{ss}}$ , while we verified that the convergence time is comparable to the one observed for the static policy.

## 7 Robotarium experiments

To further validate our control algorithm, we tested it in a real robotic scenario, using the open access *Robotarium* platform; see Pickem et al. (2017); Wilson et al. (2020) for further details. The experimental setup features 20 differential drive robots (GRITSBot Pickem et al., 2015), that can move in a 3.2 m × 2 m rectangular arena. The robots have a diameter of about 11 cm, a maximum (linear) speed of 20 cm/s, and a maximum rotational speed of about 3.6 rad/s. To cope with the limited size of the arena, distances $\left\|r_{ij}\right\|$ in (9) are doubled, while control inputs **u**_*i*_ are halved. The Robotarium implementation includes a collision avoidance safety protocol and transforms the velocity inputs (7) into appropriate acceleration control inputs for the robots. Moreover, we run an initial routine to yield an initial condition in which the agents are aggregated as much as possible at the centre, similarly to what considered in Section 5.

As a paradigmatic example, we performed a flexibility test (similarly to what done in Section 5.3.3 and reported in Figure 8). During the first 33 s, the agents reach an aggregated initial condition. Then we set *L* = 4 for *t* ∈ [33, 165), *L* = 6 for *t* ∈ [165, 297), and *L* = 4 for *t* ∈ [297, 429], ending the simulation. We used the static control law (7)–(8) and (11), and to comply with the limited size of the arena, we scaled the control gains to the values *G*_r_ = 0.8 and *G*_n_ = 0.4, selected empirically.

**FIGURE 13:**
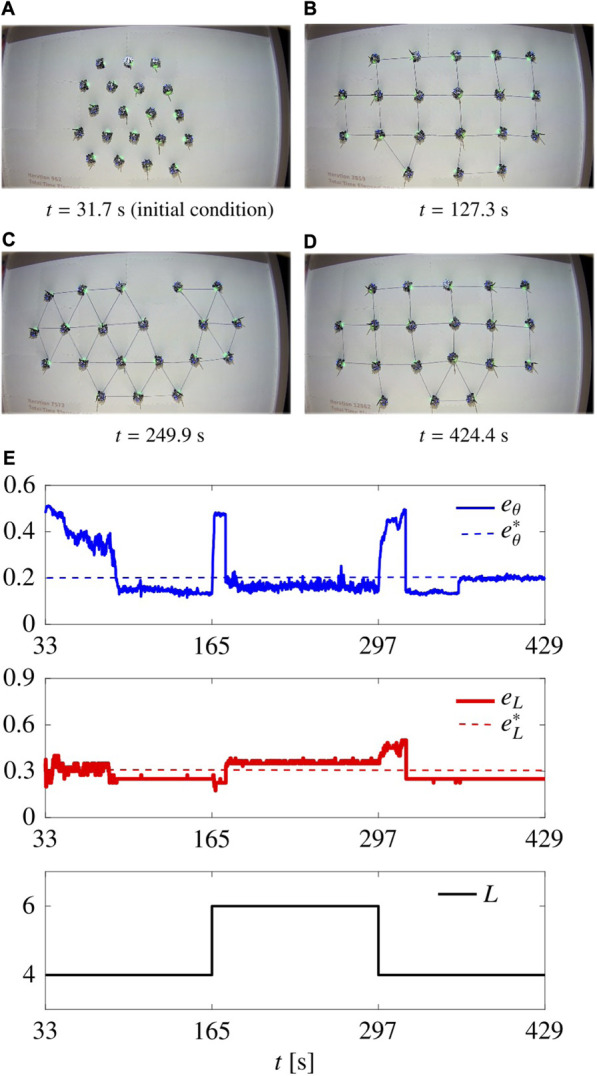
Flexibility test in Robotarium. **(A–D)** show the swarm at different time instants. **(E)** shows the time evolution of the metrics and the parameter *L*. The gains are set as $\left(G_{\text{r}},G_{\text{n}}\right)={\left(G_{\text{r}}^{*},G_{\text{n}}^{*}\right)}_{L=4}$ .

The resulting movie is available online (https://github.com/diBernardoGroup/SwarmSimPublic/tree/SwarmSimV1/Media), while representative snapshots are reported in Figure 13, with the time evolution of the metrics. The metrics qualitatively reproduce the behaviour obtained in simulation (see Figure 8). In particular, we obtain $e_{\theta }^{\text{ss}}<e_{\theta }^{*}$ , with both triangular and square patterns. On the other hand, we obtain $e_{L}^{\text{ss}}<e_{L}^{*}$ when forming square patterns, but $e_{L}^{\text{ss}}>e_{L}^{*}$ with triangular patterns; this does not mean that the pattern is not achieved, as it can be seen in Figure 13C showing the pattern is successfully achieved. This minor performance degradation is due to (i) the reduced number of agents, (ii) unmodelled dynamics of the differential-drive robots such as non-holonomic constraints and finite acceleration, and (iii) additional constraints such as the finite size of the arena and the size of the robots themselves.

## 8 Conclusion

We presented a distributed control law to solve pattern formation for the case of square and triangular lattices, based on the use of virtual forces. Our control strategy is distributed, only requires distance sensors and a compass, and does not need communication between the agents. We showed via exhaustive simulations and experiments that the strategy is effective in achieving triangular and square lattices. As a benchmark, we also compared it with the well established distance-based strategy in Spears et al. (2004), observing better performance particularly when the goal is that of achieving square lattices. Additionally, we showed that the control law is robust to failures of the agents and to noise, it is flexible to changes in the desired lattice and scalable with respect to the number of agents. We also presented a simple yet effective gain adaptation law to automatically tune the gains so as to be able to switch the goal pattern in real-time.

In the future, we plan to study analytically the stability and convergence of the control law; results in the case of triangular lattices, also for higher dimensions, were recently presented in Giusti et al. (2023). Other possible future extensions include the ability to obtain other patterns (e.g. hexagonal ones, or non-regular tilings), move in 3D environments and the synthesis of a more sophisticated adaptive law, or a reinforcement learning strategy, able to tune all the control gains at the same time.

## Data Availability

The software platform used to produce the results presented in the paper is SWARMSIMV1, which is available here: https://github.com/diBernardoGroup/SwarmSimPublic/tree/SwarmSimV1.

## Footnotes

1 Formally, G(t) is a directed graph, even though E(t) is such that the existence of (*i*, *j*) implies the existence of (*j*, *i*).

2 Regular tilings exist only for *L* ∈ {3, 4, 6}. The case *L* = 2 corresponds more trivially to a line, rather than a planar structure. The case *L* = 3, corresponding to hexagonal tilings, where vertices appear in two different spatial configurations (one with edges at angles *π*/2, 7/6 *π*, 11/6 *π*, and one with edges at angles *π*/6, 5/6 *π*, 3/2 *π*—plus an optional offset). Hence, the control strategy we propose here would need to be extended to select one or the other of the possible hexagonal configurations.

3 First order models like (6) are often used in the literature Lee and Chong (2008); Lee et al. (2010); Casteigts et al. (2012); Zhao et al. (2019). In some other works Spears et al. (2004); Sailesh et al. (2014) a second order model is used, given by $m{\ddot{x}}_{i}+\mu {\dot{x}}_{i}=u_{i}$ , where **u**_*i*_ is a force, *m* is a mass and *μ* is a viscous friction coefficient. Under the simplifying assumptions of small inertia $\left(m\|{\dot{v}}_{i}\|\ll \mu \|v_{i}\|\right)$ and *μ* = 1, the two models coincide.

4 That is, denoting with *U*([*a*, *b*]) the uniform distribution on the interval [*a*, *b*], the initial position of each agent in polar coordinates **x**_*i*_(0):= (*d*_*i*_, *ϕ*_*i*_) is obtained by independently sampling $\phi_{i}∼U\left([0,2\pi [\right)$ and *d*_*i*_ is chosen according to the probability density function $p_{l}\left(\xi \right):\left[0,r\right]\mapsto R_{\geq 0}$ defined as *p*_*l*_(*ξ*) = 2*ξ*/*r*2.

5 We also considered different deployment strategies (e.g., agents starting uniformly distributed from a larger disk or several disjoint disks) and verified that the results are qualitatively similar.

6 Simulations are performed using SwarmSimV1, a software platform we developed to simulate swarms of mobile agents. The code is available at https://github.com/diBernardoGroup/SwarmSimPublic/tree/SwarmSimV1.

## Appendix

Let us first introduce some useful notation. Given a real-valued function *x*(*t*) and a,b∈R with *a* < *b*, we introduce the *saturation* of *x*(*t*) between *a* and *b*, given by

$${\left[x\left(t\right)\right]}_{a}^{b}:=\left\{\begin{array}{c} a,\;\text{if}\;\;x\left(t\right)<a,\\ x\left(t\right),\;\text{if}\;\;a\leq x\left(t\right)\leq b,\\ b,\;\text{if}\;\;x\left(t\right)>b. \end{array}\right.$$

In Spears and Gordon (1999); Spears et al. (2004); Sailesh et al. (2014), the agent dynamics is described by

$$\left\{\begin{array}{c} {\dot{x}}_{i}=v_{i},\\ {\dot{v}}_{i}=\frac{1}{m}\left(u_{i}-\mu v_{i}\right), \end{array}\right.\;\forall i\in S,$$
(23)

where $u_{i}\in R^{2}$ is the control input, $m\in R_{>0}$ is the mass of the agent and $\mu \in R_{>0}$ is the friction damping factor. Recall that, as described in Section 4.1, under a few assumptions, (23) can be recast as (6). The control input **u**_*i*_ is given by

$$u_{i}=\overset{N}{\underset{j=1}{\sum }}f\left(\left\|r_{ij}\right\|\right)\frac{r_{ij}}{\left\|r_{ij}\right\|},$$
(24)

where *f* is a gravitational-like virtual force, given by

$$f\left(\left\|r_{ij}\right\|\right)=\left\{\begin{array}{c} {\left[G\frac{m^{2}}{\|r_{ij}{\|}^{2}}\right]}_{0}^{F_{\max}},\;\text{if }\;0\leq \|r_{ij}\|\leq R,\\ -{\left[G\frac{m^{2}}{\|r_{ij}{\|}^{2}}\right]}_{0}^{F_{\max}},\;\text{if }\;R<\|r_{ij}\|\leq 1.5R,\\ 0,\;\text{otherwise}. \end{array}\right.$$
(25)

In (25), $G,F_{\max}\in R_{\geq 0}$ are tunable control gains, and $R\in R_{>0}$ .

The control law given by (24) and (25) was showed to work for triangular lattices. To make it suitable for square patterns, a binary variable called *spin* is introduced for each agent, and the swarm is divided in two subsets, depending on the value of their spin. Then, agents with different spin aggregate at a distance of *R*, while agents with the same spin do so at a distance of $\sqrt{2}R$ . The extension to the case of hexagonal lattice is discussed in Sailesh et al. (2014) and requires communication among the agents.
